# High-throughput *in situ* cell electroporation microsystem for parallel delivery of single guide RNAs into mammalian cells

**DOI:** 10.1038/srep42512

**Published:** 2017-02-13

**Authors:** Shengtai Bian, Yicen Zhou, Yawei Hu, Jing Cheng, Xiaofang Chen, Youchun Xu, Peng Liu

**Affiliations:** 1 Department of Biomedical Engineering, School of Medicine, Collaborative Innovation Center for Diagnosis and Treatment of Infectious Diseases, Tsinghua University, Beijing, 100084, China; 2 School of Biological Science and Medical Engineering, Beihang University, Beijing, 100191, China

## Abstract

Arrayed genetic screens mediated by the CRISPR/Cas9 technology with single guide RNA (sgRNA) libraries demand a high-throughput platform capable of transfecting diverse cell types at a high efficiency in a genome-wide scale for detection and analysis of sophisticated cellular phenotypes. Here we developed a high-throughput *in situ* cell electroporation (HiCEP) microsystem which leveraged the superhydrophobic feature of the microwell array to achieve individually controlled conditions in each microwell and coupled an interdigital electrode array chip with the microwells in a modular-based scheme for highly efficient delivery of exogenous molecules into cells. Two plasmids encoding enhanced green and red fluorescent proteins (EGFP and ERFP), respectively, were successfully electroporated into attached HeLa cells on a 169-microwell array chip with transfection efficiencies of 71.6 ± 11.4% and 62.9 ± 2.7%, and a cell viability above 95%. We also successfully conducted selective electroporation of sgRNA into 293T cells expressing the Cas9 nuclease in a high-throughput manner and observed the four-fold increase of the GFP intensities due to the repair of the protein coding sequences mediated by the CRISPR/Cas9 system. This study proved that this HiCEP system has the great potential to be used for arrayed functional screens with genome-wide CRISPR libraries on hard-to-transfect cells in the future.

In the post-genome era, genetic screening has emerged as a rapid, powerful approach to annotate gene functions through evaluating phenotypical changes of cells resulted from intentional alterations of gene expressions in a pathway- or genome-wide scale^1^,^2^,^3^,^4^. Methods to achieve such gene perturbations include cDNA expression cloning^5^, RNA interference (RNAi)^4^,^6^,^7^, and more recently, clustered regularly interspaced short palindromic repeats (CRISPR)/CRISPR-associated 9 (Cas9) gene editing^1^,^2^,^8^. In general, functional screens can be conducted in either a pooled or an arrayed format^3^,^9^. While the pooled screening assay possesses the advantages of easy library preparation, relatively low cost, and no need for robotics, only simple phenotypes, such as cell proliferation or survival, can be analyzed as all the transduced cells are mixed in a single tube^2^,^10^,^11^. By contrast, since each well in a microtiter plate reagent contains cells with known genetic modifications, the arrayed gene function screening is capable of interrogating a much wider range of cellular phenotypes using more powerful detection tools, such as high-content microscopy^12^,^13^,^14^,^15^. Unfortunately, the arrayed assay is expensive in reagent synthesis and is heavily dependent on the use of liquid handling robotics. Recently, GE Pharmacon (Lafayette, CO) and ThermoFisher Scientific (Waltham, MA) have already released single guide RNA (sgRNA) libraries for arrayed CRISPR/Cas9 screening, which overcame the challenge in reagent synthesis. Hence, it becomes more imperative to increase the throughput of the arrays and lower the cost per assay by developing novel screening platforms for cell analysis.

One promising approach to overcome the drawbacks mentioned above of arrayed screens is to replace the conventional microtiter plate with a cell microarray, on which a lawn of cells is cultured on a planar slide with a spotted array of transfection reagents^16^,^17^,^18^,^19^. Cells were “reverse transfected” on each reagent spot and analyzed by scanning for phenotypical changes. Cell microarray technology is attractive because of its high throughput, low reagent consumption, and high-content readouts. However, since the chemical transfection is not applicable to many cell types, especially primary cells, more efficient and versatile cell transfection methods are highly demanded on the cell microarray platform^17^. In addition, cell clusters cultured on a microarray slide are exposed to a homogenous culture condition, resulting in the possibility of neighboring effects or cross-contamination^20^. The lack of segregation of different cell clusters also confounds the image-based analysis of phenotypic changes, resulting in high rates of false positives and negatives.

To date, many technologies have been developed to realize the delivery of exogenous molecules into living cells *in vivo* or *in vitro*, including chemical transfection^21^, viral transduction^22^, gene gun^23^, and electroporation^24^,^25^. Among them, electroporation has been widely recognized as one of the most versatile and straightforward approaches with the advantages of high transfection efficiency, minimized cell toxicity, and convenient operations^25^,^26^,^27^. Recently, electroporation has been successfully demonstrated to efficiently deliver mRNA into mouse zygotes for CRISPR/Cas9-based genome editing^28^,^29^. Electroporation can also be used for extracting genomic DNA or releasing other intracellular molecules out from cells for downstream analyses in microfabricated devices^30^,^31^,^32^. To address the need of the high throughput in genetic screenings, several groups have developed a variety of miniaturized electroporation systems by taking the advantages of micromachining and microfabrication. For instances, Guignet *et al*. developed a suspended-drop electroporation system that was coupled with a 96-well plate for high-throughput cell processing^33^. Later, Xu *et al*. developed a printed circuit board (PCB)-based suspended-drop system, in which each electroporation unit can be individually controlled^34^. Unfortunately, the throughput of these systems was difficult to increase due to the integration with conventional microtiter plates, and cells still must be in a suspended form for electroporation. Adopting the idea of combining cell microarray with microfabricated electrodes, Fujimoto *et al*. presented a microarray-based electroporation for high-throughput gene function studies^35^. In this method, cells were cultured on a gold electrode, on which siRNA was pre-spotted into a microarray. When an electrical pulse was applied across the electrode, only the cells on the spots were transfected with siRNA. Similarly, Saez’s group employed an indium-tin oxide (ITO)-coated glass slide as a substrate for spotting siRNAs^36^,^37^. They successfully demonstrated an initial RNAi gene knockdown on the device in a high-throughput *in situ* electroporation manner.

While the conventional cell microarray can be significantly improved by these microfabricated systems, several challenges are still left unaddressed. First, cells were “reverse transfected” by electroporation as reagents were electrostatically adsorbed on the substrate before cell seeding. This biomolecule delivery method is different from the conventional electroporation and may complicate the transfection process. Second, in a cell microarray, all the cells are usually cultured in a homogeneous condition, which cannot eliminate cross-contaminations among cell clusters. Third, since cells were cultured and electroporated directly on the electrodes, the changes of pH or temperature induced by electrolysis could damage cells^34^. Also, if the electrodes were fabricated using metals, the observation of cells using an inverted microscope become impossible^26^,^35^.

Previously, our group has successfully developed a novel superhydrophobic microwell array chip (SMARchip) for high-throughput cell culture and analysis^38^. Due to the repelling effect of the superhydrophobic layer to an aqueous solution, the conditions in individual microwells were completely isolated. The successful investigation of stem cell niches combining multiple chemical and mechanical cues proved that our SMARchip is an excellent platform for cell screening studies. To further extend the application of the SMARchip to genetic screens, here we coupled an electroporation chip containing an array of electrode units with the superhydrophobic microwell array to produce a high-throughput *in situ* cell electroporation (HiCEP) system. The electroporation reagents were selectively dispensed into individual microwells, in which cells were cultured. During electroporation, these two chips were aligned and held together face to face, so that each electrode unit made contact with the corresponding microwells through droplet connections. Parallel electroporation of the plasmids encoding fluorescent proteins as well as sgRNAs into cells was thoroughly tested on the HiCEP microsystem. We anticipate this high-throughput platform may play a major role in the arrayed CRISPR/Cas9-mediated genetic screening on hard-to-transfect cells in the future.

## Materials and Methods

### Electroporation devices and microfabrication

**Figure 1:**
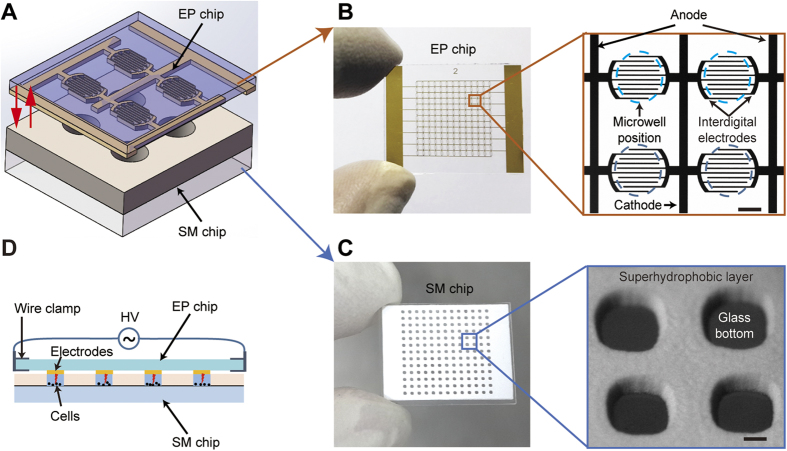
High-throughput *in situ* cell electroporation (HiCEP) microsystem. (**A**) Schematic of the HiCEP system. Two separate microchips, an electroporation chip (EP chip) and a superhydrophobic microwell chip (SM chip), were aligned and held together for on-chip electroporation. (**B**) Photo of the EP chip with a pair of major gold electrodes forming an array of 13 × 13 electrode units. The expanded view shows that each unit contains ten interdigital electrodes with a wire width of 19 μm and a spacing of 34 μm, covering a 500-μm-diameter area (blue dashed circle). (**C**) Photo of the SM chip fabricated by synthesizing an 84-μm-thick layer of superhydrophobic polymers with 500-μm-diameter holes on a piece of glass. (**D**) Working mode of the HiCEP system. The EP and the SM chips are aligned and pressed together face-to-face so that the droplets in the microwells can contact with the corresponding interdigital electrodes on the EP chip to enable *in situ* cell electroporation. Scale bar: 200 μm.

As illustrated in Fig. 1A, our high-throughput *in situ* cell electroporation (HiCEP) system consists of two separate microchips: an electroporation chip (EP chip) with microfabricated gold electrodes for applying electric pulses to cells, and a superhydrophobic microwell chip (SM chip) for cell culture and imaging. Figure 1B shows that on the EP chip, a pair of main electrodes forms an array of 13 × 13 electrode units, each of which contains ten interdigital electrodes (five pairs of cathodes and anodes) with a wire width of 19 μm and a spacing of 34 μm. Each electrode unit covers a 500-μm-diameter round area shown as a blue dashed circle in Fig. 1B. A corresponding array of 169 microwells on the SM chip is fabricated by attaching an 84-μm-thick layer of superhydrophobic polymers (poly(butyl methacrylate-co-ethylene dimethacrylate), BMA-EDMA) with 500-μm-diameter holes on a piece of glass (shown in Fig. 1C)^38^,^39^. Due to the repelling effect of the superhydrophobic polymers to aqueous solutions, a nanoliter droplet array in the microwells can spontaneously form when excess solutions outside the wells are aspirated out (Supplementary Video S1). The volume of each droplet is approximately 24 nL. As demonstrated in Fig. 1D, when these two chips are aligned and pressed together face-to-face, the droplets in the microwells of the SM chip can make contact with the corresponding electrode units on the EP chip to enable *in situ* cell electroporation on the SM chip. The detailed microfabrication methods of these two microchips can be found in the Supplementary Materials (Fig. S1). Before use, both the EP and the SM chips were soaked in 75% ethanol for 15 minutes and were exposed to UV light for 2 hours to achieve an aseptic condition.

### Cell culture and electroporation reagents

HeLa cells were obtained from the American Type Culture Collection (ATCC, Manassas, VA), and 293T cells with inserted Cas9 gene were purchased from Inovogen (Beijing, China). Both types of cells were cultured in Dulbecco’s modified Eagle’s medium (DMEM) supplemented with 10% fetal bovine serum (all from Gibco, Grand Island, NY) using a standard cell culture incubator (37 °C, 100% humidity, and 5% CO_2_). Cells were passaged every 2 or 3 days for fewer than ten passages before abandonment. Human umbilical vein endothelial cells (HUVECs) were extracted from human umbilical cord (obtained with informed consent) and cultured in Endothelial Cell Medium (ScienCell Research Laboratories, Carlsbad, CA). The plasmids used for cell electroporation are pEGFP-N1 (Clonetech, Palo Alto, CA) and EF1α-mcherry (provided by Prof. Jiakui Ji, Tsinghua University), which encode enhanced green and red fluorescent proteins (EGFP and ERFP), respectively.

### Operation procedure of cell electroporation

**Figure 2:**
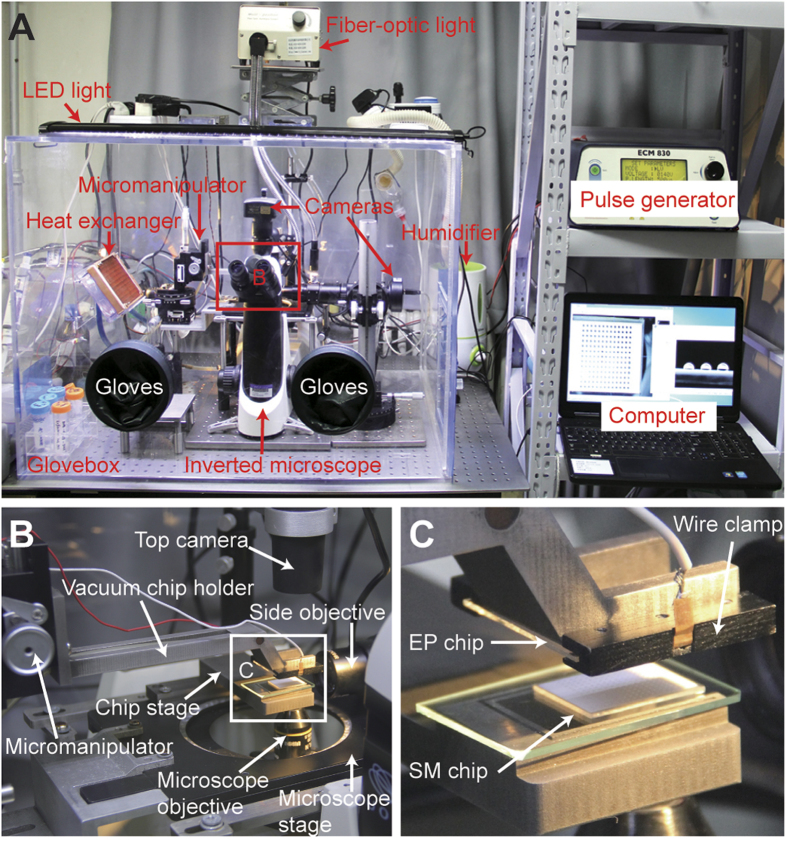
Photos of the electroporation station for the HiCEP microsystem. (**A**) Front overview of the station. An inverted microscope was set up in a custom-built glovebox, in which the humidity was maintained to prevent the droplet evaporation and the condition was sterilized using UV lamps for cell manipulation. (**B**) Modified microscope stage with a vacuum chip holder and a chip stage for the EP and the SM chips, respectively. (**C**) Expanded view of the microchips on the station. The EP chip was held by the vacuum chip holder with electrode side facing down, while the SM chip was position onto the chip stage. Both of the microchips were aligned using a custom-built micromanipulator with the aid of the CCD cameras.

**Figure 3:**
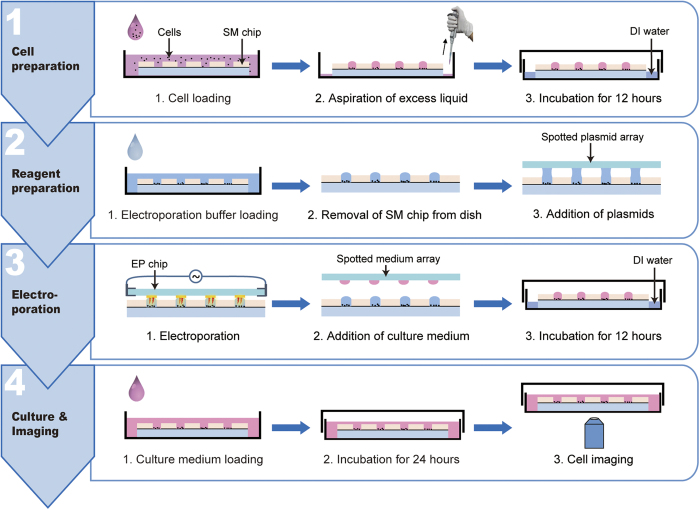
Operation procedure of high-throughput *in situ* cell electroporation. (1) Cell preparation. The cell suspension was first loaded onto the SM chip in a Petri dish, followed by an incubation of 10 minutes to let cells settle into the microwells. When the excess solution was aspirated out, a droplet array was formed on the SM chip. The cells were subsequently cultured in the SM chip for 12 hours. (2) Reagent preparation. The electroporation buffer was added into the Petri dish to exchange the solution in the microwells. Next, the plasmids were deposited into the microwells using the spot-cover method. (3) Cell electroporation. The EP chip was aligned and pressed against the SM chip on the electroporation station, and then an electric pulse was applied to the EP chip to realize the electroporation of the cells in the microwells. After that, fresh culture medium was added into the microwells by the spot-cover method. (4) Culture and imaging. The SM chip was placed into the Petri dish and the medium was changed every 12 hours for 24-hour cell culture. Finally, the Petri dish was mounted onto an inverted fluorescence microscope for cell imaging.

The on-chip cell electroporation was conducted in a custom-built electroporation station (shown in Fig. 2 and Supplementary Materials), in which the relative humidity was maintained above 90% with a humidifier and the equipment was sterilized with UV lamps. As illustrated in Fig. 3 the operation process of cell electroporation performed on the HiCEP system includes four major steps: cell preparation, reagent preparation, cell electroporation, and culture & imaging.

1. Cell preparation. First, microwells were first filled with PBS by dripping PBS onto the chip with a 10-mL stripette, which was held ~20 cm above the chip to expel the air out of the microwells. Then, the SM chip was put into a Petri dish and submerged into 5-mL DMEM for 10 min. This incubation time is sufficient to exchange the PBS with culture medium in the microwells. After that, the excess DMEM was aspirated out, resulting in a droplet array in the microwells due to the superhydrophobic polymers on the top surface of the chip. Next, a 5-mL monodisperse cell suspension at a density of 2.5–5 × 10^5 ^cells/mL was prepared and loaded into the Petri dish, submerging the entire chip for 10 minutes to allow cells to settle into the microwells. After the excess cell suspension had been aspirated, the droplet array with cells was formed again. Additional 1-mL DI water was pipetted into the dish surrounding the chip to prevent any droplet evaporation during the following 12-hour culture in a cell incubator.
2. Reagent preparation. In the reagent preparation process, a 5-mL low osmotic electroporation buffer (EP buffer, pH 7.4, conductivity 3.5 mS/cm at 25 °C) containing 25 mM KCl, 0.3 mM KH_2_PO_4_, 0.85 mM K_2_HPO_4_, and 36 mM myo-inositol was first added into the Petri dish to submerge the SM chip completely. After a 10-min incubation, excess electroporation buffer was removed, and the solution in the microwells was completely exchanged to the EP buffer. Since the cells attached to the bottom of the microwells and no agitation was needed for the buffer exchange, cells remained within the microwells. Next, to selectively deliver plasmids into each microwell, we employed a standard microarray spotter (PersonalArrayer 16, CapitalBio, Beijing, China) to print a plasmid array on a hydrophobic glass slide according to the microwell dimensions. The volume of each spot on the slide is about 32 nL, which were prevented from dry-out using a humidifier in the microarray spotter during the spotting process. After that, the SM chip was positioned onto the chip stage in the glovebox, and the plasmid slide was held by the vacuum chip holder upside down. By aligning and covering this plasmid slide onto the SM chip with the aid of a micromanipulator and a CCD camera (shown in Fig. 2), the spotted plasmids were delivered into the microwells through a droplet-to-droplet contact without any cross-contamination between adjacent microwells (Supplementary Videos S2 and S3).
3. Cell electroporation. The EP chip was connected to the pulse generator using a pair of chip clamps and then held by the vacuum chip holder with the electrode side facing down. With the aid of the CCD camera over the chips, the electrode units were precisely aligned to the corresponding microwells. Then, the EP chip was pressed against the SM chip slightly to achieve a flush contact between these two chips. A square wave signal with an amplitude of 80 V and a duration of 0.5 ms was applied to realize the electroporation of the cells in the microwells. After electroporation, the EP buffer in the microwells was diluted with fresh culture medium by covering a spotted culture medium array (32 nL/spot) to the SM chip. The transfected cells were then cultured for 12 hours in droplets to let the electroporation process complete.
4. Culture and imaging. After the cell culture in droplets, the 5-mL fresh culture medium was added into the Petri dish to submerge the SM Chip completely. Since the volume of the microwells is only 24 nL, the solution within the microwells was diluted over 10^5^ fold. After 10-min incubation, the medium was aspirated out, and another 5-mL fresh medium was added again. This simple washing step can ensure the complete washout of any un-electroporated plasmids. The cells were further cultured in an incubator for additional 24 hours either in the droplet or the chip-submerging status. In the last step, the Petri dish was mounted onto an inverted fluorescence microscope (IX71, Olympus, Tokyo, Japan) equipped with a CCD camera (Clara, Andor, Belfast, Northern Ireland) to image the cells. The entire SM chip was also imaged using a stereo microscope (Axio Zoom.V16, Zeiss, Oberkochen, Germany) with an Andor camera (Zyla 4.0 sCMOS).

### Simulation of electric field distribution

The distribution of electric fields within individual microwells was simulated using a finite element modeling software (COMSOL 4.2 with AC/DC module, COMSOL AB, Burlington, MA). The model geometry was simplified to a cross-sectional slice with dimensions of 213 × 84 × 10 μm, which was perpendicular to the interdigital electrodes along the diameter of the microwells. The dimensions of the electrodes were set to 19 μm wide, 2 μm high, and 10 μm long, and the distance between the electrodes was 34 μm. The conductivity of the electroporation buffer was set to 3.5 mS/cm. The relative permittivities of water and the gold electrodes were 81, and 1, respectively. The amplitude of the electric signal applied to the electrodes was 80 V.

### Optimization and evaluation of electroporation efficiency

To expedite the optimization of the electroporation parameters, we fabricated a simplified PDMS (polydimethylsiloxane) macro-well chip for cell electroporation. As shown in Fig. S2, a piece of PDMS membrane with four 5-mm-diameter punched holes were permanently bonded to a glass slide using oxygen plasma. The thickness of this PDMS layer is the same as that of the superhydrophobic layer on the SM chip so that the electric fields on the glass bottoms of both the devices are similar. HeLa cells were first seeded into this macro-well chip and cultured for 12 hours to reach 70% confluence. After the culture medium was changed to the electroporation buffer containing pEGFP-N1 plasmids, the EP chip was covered to the wells, and a series of voltages ranging from 20 to 100 V were applied to the cells. After 24 hours of cell culture, the transfected cells underneath the electrode units were enumerated using the IX71 fluorescent microscope to calculate the electroporation efficiencies.

Following the optimization, an on-chip cell electroporation of HeLa cells with pEGFP-N1 plasmids was conducted following the protocol described above. Prior to imaging, the cells were stained with propidium iodide (PI, Sigma-Aldrich, St Louis, MO) following the manufacturer’s instruction. After imaging all the microwells, the cells were treated with trypsin/EDTA solution at 37 °C for 5 minutes and then collected for flow cytometer analysis (FACS Calibur^TM^, BD Biosciences, East Rutherford, NJ). Both the electroporation of HeLa cells with PI and the electroporation of HUVECs with pEGFP-N1 plasmids were conducted under the same condition and were quantitated using the flow cytometer.

### Individually controlled electroporation in microwells

Both the pEGFP-N1 (500 ng/μL) and the EF1α-mcherry plasmids (500 ng/μL) diluted in the EP buffer were employed to transfect HeLa cells on the chip. The electroporation patterns were spotted onto glass slides using the microarray spotter. The transfected cell and the total cell numbers in each microwell were counted manually using the fluorescent and the bright-field images, respectively.

To demonstrate the CRISPR/Cas9-mediated gene editing, we purchased pTYNE plasmids which contain a mutant EGFP sequence and sgRNA plasmids (pGR-Hygro-P for EGFP sequence repair and pGR-Hygro-N for negative control) from Inovogen (shown in Fig. S3). The 293T cells with the Cas9 gene were first transfected with pTYNE using Lipofectamine^®^ 2000 (ThermoFisher) according to the manufacture’s instruction. Twenty-four hours after transfection, the cells were seeded into the SM chip at a density of ~50 cells/microwell, followed by selective electroporation of pGR-Hygro-P and pGR-Hygro-N plasmids in an alternate line format with a voltage of 60 V next day. Images of the cells were taken 24 hours after electroporation. To further verify the repair of the EGFP sequence, transfected cells were collected from the microchip. The fragments were amplified out using a pair of primers (pTYNE-F: TGGGAGGTCTATATAAGCAGAG, pTYNE-R: CGTCGCCGTCCAGCT CGACCAG), and then analyzed by Sanger sequencing.

## Results

### Simulation of electric field distribution in microwells

**Figure 4:**
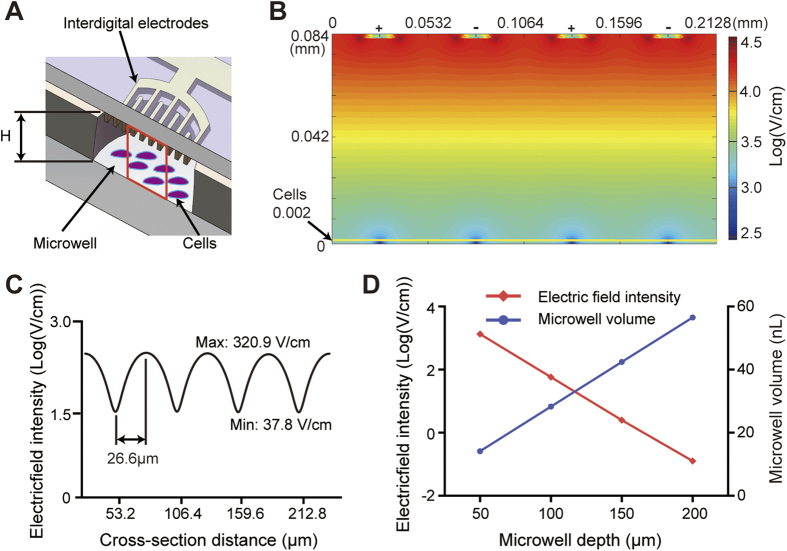
Simulation of the electric field distribution in a microwell. (**A**) Cross-sectional view of the microwell covered with the electrode unit. The red square indicates the simulated area, which is a cross-sectional slice with dimensions of 213 × 84 × 10 μm. (**B**) Color contour plot of the simulated electric field distribution when an 80 V pulse is applied to the electrodes. (**C**) Distribution of the electric field along the yellow line. The yellow line indicated in (**B**) is 2 μm above the microwell bottom. A sinusoidal distribution is obtained with a maximum valve of 320.9 V/cm and a minimum valve of 37.8 V/cm. (**D**) Maximum electric field intensity on the yellow line and volume of the microwell as a function of the depth of the microwell.

To understand the distribution of electric fields within the microwells, we employed a finite element modeling software (COMSOL 4.2 with AC/DC module) to simulate the electric field intensities on the microchip. Due to the symmetrical nature of the microwells and the electrodes, we simplified the geometry to a cross-sectional slice perpendicular to the interdigital electrodes along the diameter of the microwells (red square shown in Fig. 4A). The dimensions of this slice were set to 213 × 84 × 10 μm to reduce the calculations. Accordingly, total two pairs of the interdigital electrodes (two anodes and two cathodes arranged alternately) are included into the simulation model. As demonstrated in Fig. 4B, when 80 V is applied between two adjacent electrodes, the electric field near the corner of the electrode can reach up to 10^5^ V/cm, while the electric field drops abruptly to around 10 V/cm on the bottom of the microwell. Here we assume the electroporation occurs on the top side of the cells, which is about 2 μm above the bottom (shown as the yellow line in Fig. 4B). The electric field along the yellow line is distributed as a sinusoidal curve with a maximum valve of 320.9 V/cm and a minimum valve of 37.8 V/cm illustrated in Fig. 4C. This maximum electric field is in the range of the widely accepted voltage for electroporation reported previously^36^,^40^,^41^. Since the distance between the trough and the crest is only 26.6 μm, which is usually smaller than the size of an adherent cell spreading out on a surface, we found all the cells (HeLa and HUVEC) on the bottom were transfected uniformly (shown in the following sections). For cells with a size smaller than 26 μm, the distance between adjacent electrodes could be shortened accordingly.

Although the electrodes and the cells were completely separated in our system, we still wanted to minimize the electric field near the electrodes while providing an adequate electric strength for electroporation on the bottom of microwells, as the too high electric field will induce a strong electrolysis and generate bubbles on the electrodes. This apparently requires that we reduce the depth of the microwell to place the electrodes closely to the cells. Unfortunately, the microwell volume decreases with the depth, causing troubles in cell culture and chip operations due to accelerated evaporation of tiny droplets. As shown in Fig. 4D, when a voltage of 80 V was applied, the maximum electric field for electroporation on the bottom was increased from ~10 to 1000 V/cm when the depth of the microwell was decreased from 200 to 50 μm. Meanwhile, the volume of the microwell was decreased from 58 to 12 nL. From our experience (data not shown), it is extremely difficult to maintain droplets smaller than 20 nL in the microwells during the entire operation. Therefore, we fabricated the depth of the microwell to 84 μm, resulting in a microwell volume of 24 nL which is above the volume limit. With this depth, we then optimized the applied voltage in the following experiments to obtain the maximum electroporation efficiency.

### Optimization of Electroporation

**Figure 5:**
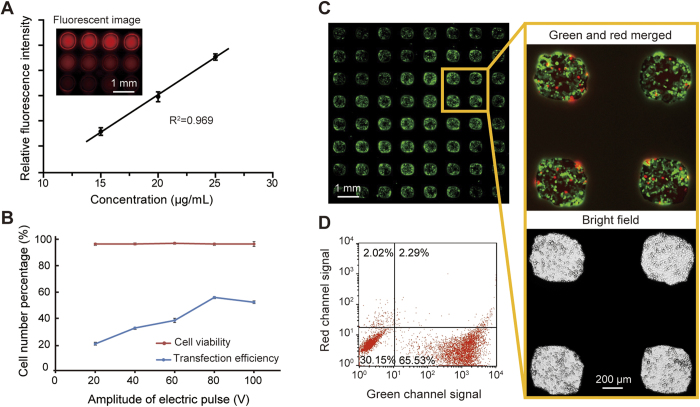
Optimization and evaluation of cell electroporation performed on the HiCEP. (**A**) Image of the microwells with various concentrations of Rhodamine B delivered using the spot-cover method. A linear relationship between the Rhodamine B concentration and the fluorescence intensity was obtained with an R^2^ equal to 0.969 (n = 8). (**B**) Optimization of the amplitude of the electric pulse applied to the electrodes. At the voltage of 80 V, the transfection efficiency reached the maximum value of 58.1%, while the cell viability is slightly decreased with the increase of the voltage. (**C**) High-throughput electroporation of HeLa cells. The average transfection efficiency is 58.4 ± 9.3%, and the percentage of the dead cells is 2.0 ± 0.9% (n = 8). (**D**) Flow cytometry result of the transfected cells recovered from the SM chip after electroporation. The overall electroporation efficiency is 65.5%, and the percentage of dead cells is 4.3%. All data are shown as the mean ± standard deviation.

The precise delivery of reagents into individual microwells without cross-contamination is the prerequisite for high-throughput cell electroporation where each microwell contains different transfected biomolecules. Although the spot-cover method had been proved effective in our previous work^38^, we tested the reagent delivery again in the HiCEP system as the structure of the superhydrophobic microwell chip was changed. In this experiment, we spotted Rhodamine B solutions with concentrations ranging from 15–25 μg/mL onto a glass slide using a microarray spotter. After that, this slide was aligned and covered to the SM chip to deposit the spotted Rhodamine B into the microwells. Figure 5A demonstrates that the fluorescence intensities of the microwells measured after the reagent delivery are linearly proportional to the concentrations of the spotted solutions with an R^2^ equal to 0.969. This linearity result not only verified the quantitative reagent delivery in our system but also proved that the cross-contamination among microwells could be prevented completely.

Many parameters can affect the outcome of cell electroporation, including cell seeding density, electroporation buffer composition, plasmid concentration, electric pulse parameters, etc. Our previous studies on microfabricated electroporation systems have successfully demonstrated up to 90% transfection efficiencies for various cell types under optimized cell-specific electroporation conditions^42^. For example, HeLa cells can be transfected with pEGFP-N1 plasmids at a ~80% efficiency using a single 0.5-ms electric pulse. To expedite the optimization process of our HiCEP system, we translated most of the operation parameters to our new setup, such as electroporation buffer, cell and reagent preparations. However, due to the structural changes of the electroporation setup, the voltage applied to the electrodes need further optimization to generate an appropriate electric field on the cell surface. The simulation of the electric field distribution presented above has estimated that an electric field of 320 V/cm can be obtained by applying 80 V to the interdigital electrodes. Therefore, based on the initial calculation, we conducted cell electroporation tests by applying a series of voltages from 20 to 100 V with a step of 20 V on a simplified PDMS macro-well chip, which has the same well depth as that of the SM chip. We fixed the duration and the number of the electric pulses used in our system. As shown in Fig. 5B, the voltage of 80 V produced the best efficiency (58.1%) of cell electroporation, while the cell viability was slightly decreased with the increase of the electric field. Also, we didn’t observe any bubbles generated on the electrodes as the applied voltage was only 80 V.

Following the optimization, we conducted a complete cell electroporation on the HiCEP system. In this experiment, pEGFP-N1 plasmids were delivered into the individual microwells using the spot-cover method and the final concentration of the plasmids in the microwell was diluted to about 233 ng/μL. Following the electroporation process, the cells were stained with propidium iodide to check the cell viability. The entire microwell array was imaged using the fluorescent stereo microscope, and the total and the fluorescent cell numbers in each microwell were manually counted to access the electroporation efficiency and the cell viability. As demonstrated in Fig. 5C, the average electroporation efficiency in the microwells was determined to be approximately 58.4% with a standard deviation of 9.3% (n = 8). The percentage of the dead cells is about 2.0 ± 0.9% (n = 8). Due to cell crowding, it is difficult to count the cell numbers accurately. Therefore, we collected all the cells from the microchip using trypsin treatment, and then analyzed all the cells on a flow cytometry. The data in Fig. 5D shows that the overall electroporation efficiency is 65.5%, and the dead cell percentage is 4.3%, both of which are close to the cell counting results. As shown in Fig. S3, propidium iodide can also be delivered into HeLa cells with an electroporation efficiency of 47.25% and a cell viability of 91.9%. In addition, since one of the most important advantages provided by electroporation is the capability of transfecting primary cells, we conducted the electroporation of the pEGFP-N1 plasmid into primary HUVECs on the chip. An efficiency of 40.6% and a cell viability of 90.2% were obtained (Fig. S4). These tests established the feasibility of performing a high-throughput cell electroporation on the HiCEP microsystem.

### High-throughput electroporation in an individually controlled manner

**Figure 6:**
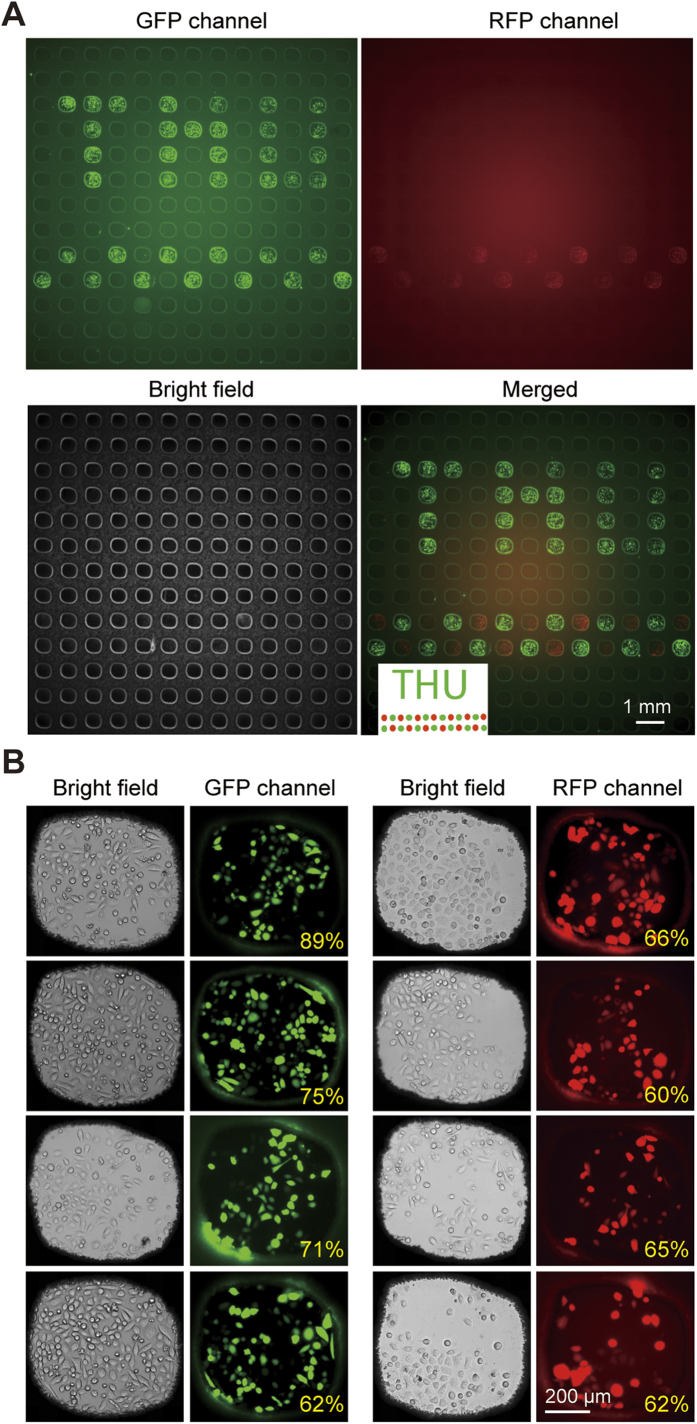
High-throughput electroporation on the HiCEP microsystem. (**A**) Fluorescent and bright-field micrographs showing a “THU” pattern were obtained by selectively transfected HeLa cells with EGFP and ERFP plasmids. The insert is the design of the “THU” pattern. (**B**) Typical fluorescent micrographs of the transfected cells in microwells. The transfection efficiencies of the EGFP and the ERFP plasmids were determined to be 71.6 ± 11.4% and 62.9 ± 2.7%, respectively (n = 8). All data are shown as the mean ± standard deviation.

To demonstrate the capability of performing electroporation of different reagents in microwells simultaneously, we employed two fluorescent protein plasmids, pEGFP-N1 and the EF1α-mcherry, to transfect HeLa cells on a single device. These two plasmids were spotted onto a glass slide to form a designed “THU” pattern, followed by aligning and covering this slide onto an SM chip with pre-seeded cells. As illustrated in Fig. 6, plasmids were delivered into specific microwells, and a colorful “THU” pattern was generated by transfected cells after electroporation. The merged image shows that there is no cross-contamination between adjacent microwells. The transfection efficiencies of the EGFP and the ERFP plasmids were determined to be 71.6 ± 11.4% and 62.9 ± 2.7%, respectively, by calculating from the images of individual microwells (n = 8). These results illustrated that the selective gene electroporation could be achieved on this platform without any detectable cross-contamination among microwells.

### Modulation of gene expression with CRISPR/Cas9

**Figure 7:**
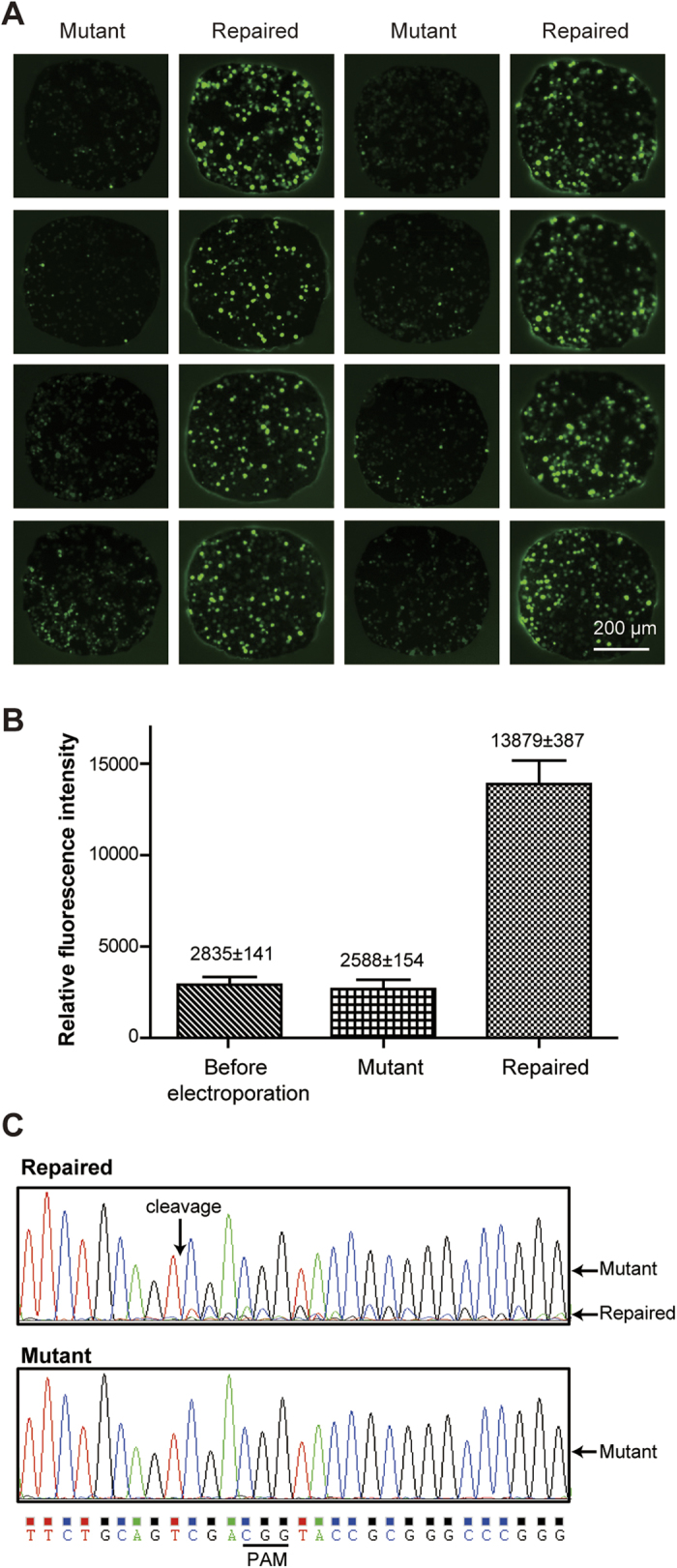
Selective delivery of single guide RNA into 293T cells on the HiCEP system. (**A**) Typical fluorescent micrographs of Cas9-expressing 293T cells transfected with different sgRNAs in parallel. Cells with pGR-Hygro-P plasmids (repaired) demonstrated a higher level of GFP fluorescent intensities than those of cells with pGR-Hygro-N (mutant), illustrating the effectiveness of the CRISPR/Cas9 system on the chip. (**B**) The difference of fluorescence intensities between these two groups is more than 4 times (n = 8, two-tailed, unpaired t-test, p < 0.001). No change was obtained before and after electroporation in the mutant group. (**C**) Sanger sequencing results of the repaired and the mutant sequences. Mixed sequences were obtained from the repaired group, suggesting an indel was successfully introduced into the plasmids by the CRISPR/Cas9 system.

A 293T cell line with the Cas9 gene inserted in the genome was employed to verify the capability of the electroporation system for the sgRNA delivery in CRISPR/Cas9 gene editing. The cells were first transfected with the pTYNE plasmid which has an EGFP coding sequence located 112-bp and 122-bp downstream from two start codons, respectively (Fig. S5). The EGFP gene cannot express efficiently due to the frameshift. Thus a very low level of green fluorescence was detected after the transfection. A guide RNA targeting the sequence between the EGFP and the start codons was then delivered into the cells using the electroporation system. After the DNA break mediated by the Cas9 protein and the guide RNA, DNA was repaired by the error-prone non-homologous end joining (NHEJ) which often leads to short insertions or deletions, thus shifts the reading frame of EGFP so that the protein can express efficiently. In this experiment, the 293T cells expressing the Cas9 protein were first transfected with pTYNE plasmids off-chip and then seeded into an SM chip. Single guide RNA plasmids, pGR-Hygro-P and pGR-Hygro-N, were deposited into microwells in an alternate line format using the spot-cover method, and then electroporated into cells on the chip. As shown in Fig. 7, after electroporation of the sgRNAs, the green fluorescence levels of the cells with pGR-Hygro-P plasmids were significantly increased around four times, while cells electroporated with pGR-Hygro-N had minor changes. To further verify the repair of the EGFP sequences, 293T cells electroporated with pGR-Hygro-P and pGR-Hygro-N, respectively, were collected from the microchips and were subjected to verification of Sanger sequencing. As shown in Fig. 7C, the mixed sequences after the cleavage site obtained from the repaired group suggested the success of the on-chip electroporation with the sgRNAs. These results demonstrated that our electroporation device could deliver sgRNA into cells efficiently in a high-throughput manner.

## Discussion

Arrayed high-throughput genetic screening mediated by the CRISPR/Cas9 system will play an important role in the quest of deciphering the functions of the entire human genome^3^. However, the current screen methodology heavily relies on the use of 96- or 384-well microtiter plates that are handled by a robotic system. Such a screening platform is expensive, consumes relatively large amounts of reagents, and has limited potential for further throughput improvement. Moreover, the lack of efficient, versatile transfection approaches limited its applications in hard-to-transfect cells, such as primary cells. Although viral transduction can be employed for delivering biomolecules, the virus production in a large scale is expensive, labor-intensive, and often causes safety concerns. In contrast, cell electroporation is much easier regarding reagent preparations and operations and is efficient for all kinds of cell types after necessary parameter optimizations. Thus, the integration of cell electroporation with a high-throughput cell handling system should have great potential to advance the performance of gene function screens.

As demonstrated in this study, our HiCEP microsystem provides several unique advantages that make the system an excellent platform for functional genomic studies. First, due to the superhydrophobic surface of the SM chip, the physical isolation of the conditions in microwells can be facilely achieved on a single device. Thus, unlike any other microarray-like systems, where transfection reagents were electrostatically adsorbed onto the substrate surfaces^35^,^37^, our superhydrophobic microwell chip allows us to keep the transfection reagents in a soluble form in the microwells. The electroporation performed in a liquid format is more like that in the conventional setup and should be able to provide a higher transfection efficiency for more types of cells without the interference from the liquid-solid interface. Second, since the electrodes are located above cells with a distance equal to the depth of the microwells (84 μm) in our HiCEP system, the changes of pH and temperature that often occur on the electrode surfaces have a minimal effect to the cells growing on the bottoms. As a result, the cell viabilities after electroporation were usually over 90% obtained in our system. This design also enables us to transfect cells in their adherent status instead of suspended, probably reducing the disturbance to normal cellular processes^25^. Third, as no electrodes are on the SM chip, the design and fabrication of this device become much easier. To lower the cost of the SM chip, we developed a new fabrication method to directly polymerize a superhydrophobic layer with a microwell pattern onto a blank glass slide using a reusable plastic mold. Compared to the SMARchip demonstrated previously^38^, this disposable SM chip is simple, inexpensive, and can be fabricated in less than 20 min while still providing a similar performance for high-throughput cell culture and analysis. Furthermore, the more expensive EP chip can be used for multiple times with a simple wash in between, so that the cost of each assay can be significantly reduced.

CRISPR/Cas9-mediated gene function studies have attracted tremendous attention due to its high efficiency and accuracy in the modulation of transcriptional activities. To the best of our knowledge, our study is the first report to demonstrate the electroporation of sgRNA into cells in a microfabricated high-throughput electroporation system. Although we only presented a small-scale device with 169 microwells in the current study, a high-throughput array with hundreds or thousands of microwells can be readily produced just like that shown in our previous work^38^. Also, we believe the HiCEP system can work for most of the cell types, as a variety of cell types had been successfully electroporated previously^42^, and the current study with HeLa cells proved that the operation parameters obtained before could be translated into the HiCEP system. In the current study, 293T cells expressing Cas9 proteins were employed to test the CRISPR/Cas9-mediated gene editing on the chip. In the future, if cells without Cas9 are used, we can first transfect the cells with Cas9 plasmids using conventional methods in a large batch, followed by the delivery of multiple sgRNAs into cells on the HiCEP system. In summary, the proof-of-concept study of transfecting cells with plasmids and sgRNA validated the feasibility of performing a large-scale genetic screening with a sgRNA library on our system in the future.

## Additional Information

**How to cite this article**: Bian, S. *et al*. High-throughput *in situ* cell electroporation microsystem for parallel delivery of single guide RNAs into mammalian cells. *Sci. Rep.* **7**, 42512; doi: 10.1038/srep42512 (2017).

**Publisher's note:** Springer Nature remains neutral with regard to jurisdictional claims in published maps and institutional affiliations.

## Supplementary Material

Supplementary Materials

Supplementary Video 1

Supplementary Video 2

Supplementary Video 3
