# Supplementary material for: High-throughput in situ cell electroporation microsystem for parallel delivery of single guide RNAs into mammalian cells
Source: Sci Rep. 2017 Feb 13;7:42512. doi: 10.1038/srep42512 (PMC5304186; doi:10.1038/srep42512)
Supplement: Supplementary Materials [file srep42512-s1.pdf]

Supplementary Information

High-throughput *in situ* cell electroporation microsystem for parallel delivery of single guide RNAs into mammalian cells

Shengtai Bian,[1,#] Yicen Zhou,[1,#] Yawei Hu,[1] Jing Cheng,[1] Xiaofang Chen,[2,*] Youchun Xu,[1,*] Peng Liu[1,*]

[1] Department of Biomedical Engineering, School of Medicine, Collaborative Innovation Center for Diagnosis and Treatment of Infectious Diseases, Tsinghua University, Beijing, 100084, China

[2] School of Biological Science and Medical Engineering, Beihang University, Beijing, 100191, China

[#] Joint first authors with equal contributions

* Joint corresponding authors: Peng Liu, Department of Biomedical Engineering, School of Medicine, Tsinghua University, Haidian District, Beijing, 100084, China. Phone: +86-10-62798732, fax: +86-10-62798732, email: pliu@tsinghua.edu.cn

Youchun Xu, Department of Biomedical Engineering, School of Medicine, Tsinghua University, Haidian District, Beijing, 100084, China. Phone: +86-10-62798732, fax: +86-10-62798732, email: xyc2012@tsinghua.edu.cn

Xiaofang Chen, School of Biological Science and Medical Engineering, Beihang University, Haidian District, Beijing, 100191, China. Phone: +86-10-82315554, fax: +86-10-82315554, email: xfchen@buaa.edu.cn

Supplementary materials and methods

Microchip fabrication

The electroporation chip was microfabricated using a standard thin-film photolithographic method. Briefly, a double polished 4” glass wafer (Borofloat® 33, Schott AG, Mainz, Germany) was first coated with 200-Å Ti and 2000-Å Au layers by sputtering. The metal layer was then patterned with a positive photoresist PR-1500A (Futurrex, Franklin, NJ) and etched to form electrodes. This EP chip can be reused for multiple times after simply washing with acetone and DI water. The superhydrophobic microwell chip was fabricated following the procedure shown in Fig. S1. First, a polished glass slide (Borofloat® 33) was cleaned with a piranha solution (H_2SO_4:H_2O_2=3:1) for 30 minutes to activate the surface. Then, the slide was salinized with 3-(trimethoxysilyl)propyl methacrylate (Sigma-Aldrich, St Louis, MO), rinsed with acetone, and dried completely. Meanwhile, a poly(methyl methacrylate) (PMMA) mold with an array of posts (500 µm diameter, 100 µm high, 500 µm pitch) was milled using a numerically controlled milling machine (MODEL 5410, Sherline, Vista, CA). The prepared glass slide and the PMMA mold were assembled together and held tightly with a custom-made clamp holder. After that, a superhydrophobic polymer premix (24% wt butyl methacrylate (BMA), 16% wt ethylene dimethacrylate (EDMA), 60% wt 1-decanol, and 1% wt 2,2-dimethoxy-2-phenylacetophenone (DMPAP), all from Sigma-Aldrich) was slowly loaded into the gap between the slide and the mold without introducing bubbles. After exposure in a UV cross-linker oven (302nm, CL-1000, UVP, CA) for 15 minutes, the assembly were slowly pried open. The polymerized BMA-EDMA layer with holes remained on the glass slide, forming an array of microwells with blank glass bottoms and superhydrophobic polymer walls. Prior to use, both the EP and the SM chips were soaked in 75% ethanol for 15 minutes, and were exposed to UV light for 2

hours in order to achieve an aseptic condition.

Custom-built electroporation station

The high-throughput *in situ* cell electroporation microsystem was operated in a custom-built glovebox in order to prevent the evaporation of the nano-liter droplets and to provide a sterilized condition during electroporation. As illustrated in Fig. 2A, an inverted microscope (DSZ2000X, Chongqing COIC, Chongqing, China) with a CCD camera (UHCCD05 100KPA, Touptek, Hangzhou, China) was setup in a sealed plexiglass glovebox, on the front panel of which a pair of gloves were installed for access. A humidifier was connected to the glovebox to maintain the relative humidity above 90% during operation. Fig. 2B shows that an assembled micromanipulator (containing XYZ, rotation, and tilt stages, Daheng, Beijing, China) with a vacuum chip holder for holding the EP chip and a chip stage for supporting the SM chip were installed on the microscope stage. Both the micromanipulator and the chip stage can move along with the microscope stage so that the entire chips can be observed from the bottom using the microscope objective (4X and 10X). As shown in Fig. 2B, a mini CCD camera was installed over the microchips for aiding the alignment of these two chips. For observing from the side to ensure the contact of droplets to the EP chip, a horizontal objective (ML-Z07545HRD, Moritex, Saitama, Japan) coupled with a CCD camera (MD900, Shangguang, Shanghai, China) was setup beside the microscope. All the CCD cameras were linked to a laptop outside the box for display. To ensure an aseptic condition in the glovebox, two UV lamps were employed to sterilize the whole system before and after each run for an hour. In addition, a LED white light as well as a fiber-optic light shown in Fig. 2A was installed for system illumination during operations. An electric pulse generator (ECM830, BTX Harvard Apparatus,

Holliston, MA) placed outside the box was connected to the electrodes on the EP chip through electrical wires and a pair of custom-built chip clamps (Fig. 2C).

Supplementary figures

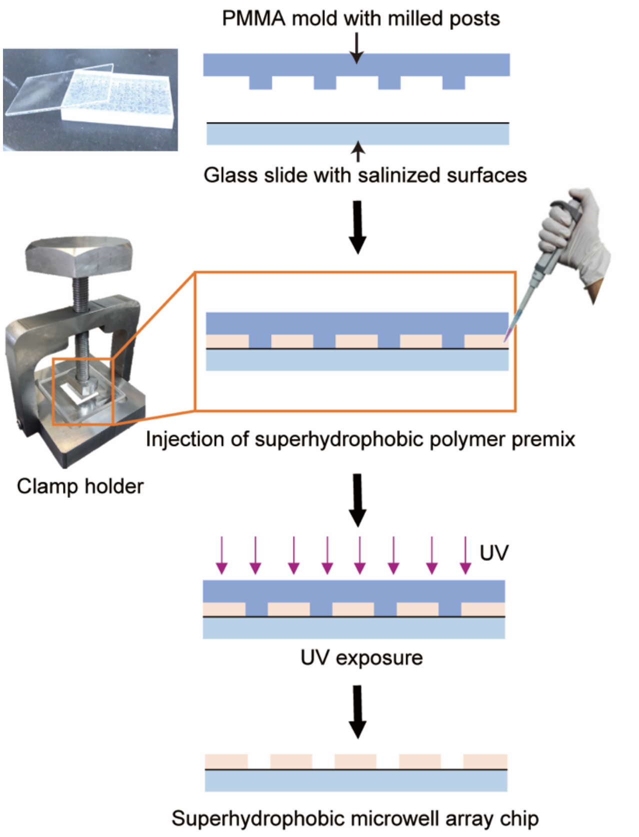

Figure S1. Microfabrication procedure of the superhydrophobic microwell chip. The fabrication employed a glass slide salinized with 3-(trimethoxysilyl)propyl methacrylate, and a poly(methyl methacrylate) (PMMA) mold with an array of posts (500 µm diameter, 100 µm high, 500 µm pitch), both of which were assembled together and held tightly with a custom-made clamp holder. A superhydrophobic polymer premix was loaded into the gap between the slide and the mold, and then exposed to UV light for 15 minutes. After the assembly were pried open, a superhydrophobic microwell chip was formed by the polymerized BMA-EDMA layer and the glass slide.

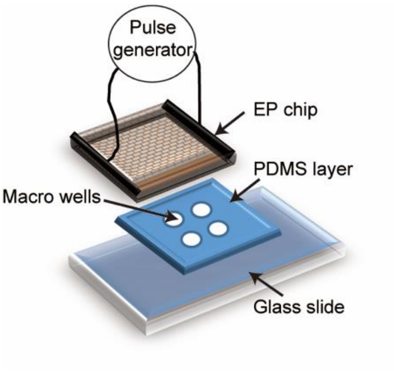

Figure S2. Schematic of the PDMS macro-well chip. A piece of 100-μm-thick PDMS membrane with four 5-mm-diameter holes were bonded to a glass slide to form the PDMS macro-well chip. The electroporation chip connected to a pulse generator was pressed against the wells to enable the cell electroporation.

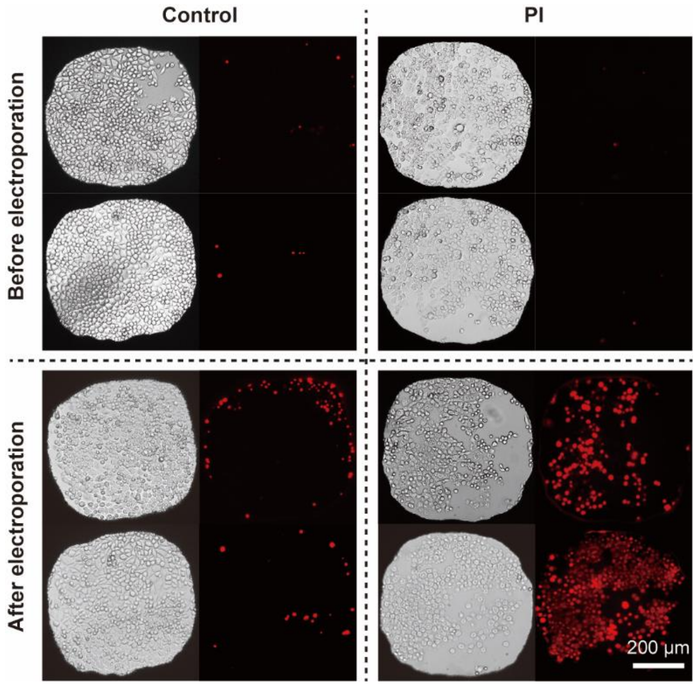

Figure S3. On-chip electroporation of HeLa cells with propidium iodide (PI). Control group: HeLa cells underwent electroporation without PI. After electroporation, cells were stained with PI to show the cell viability. PI group: HeLa cells were electroporated with PI, showing the electroporation efficiency. An electroporation efficiency of 47.25% and a cell viability of 91.9% were obtained.

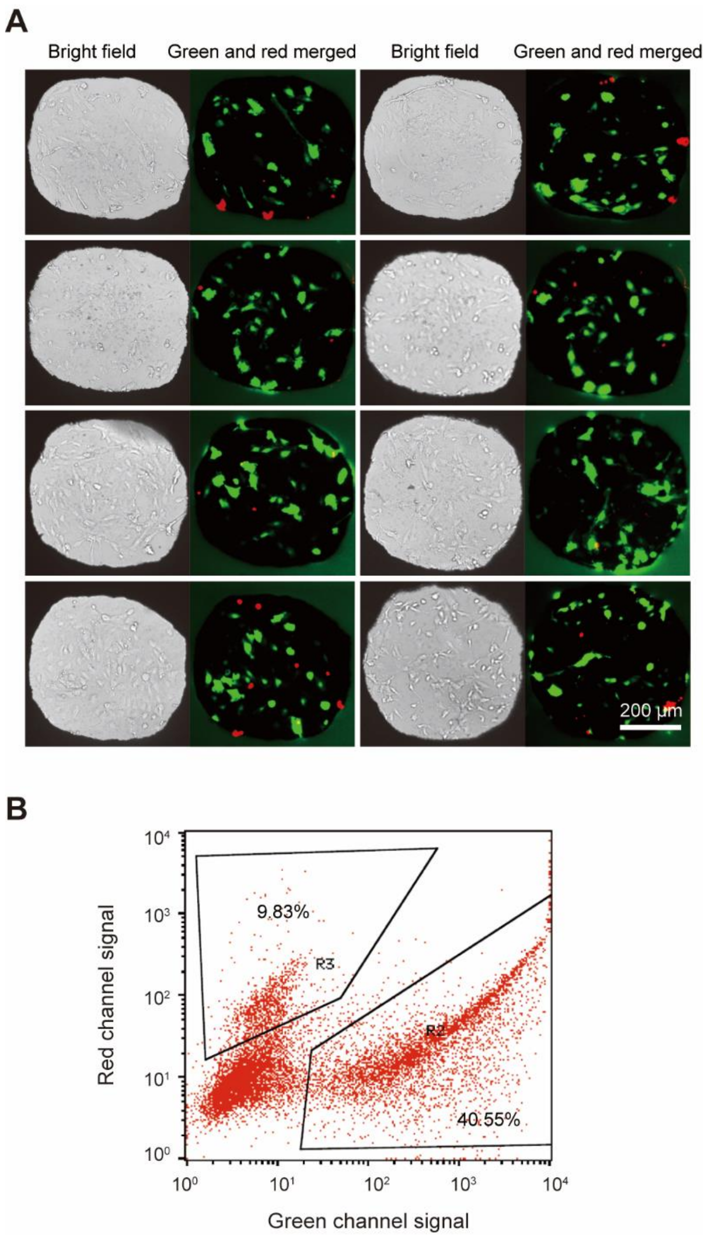

Figure S4. On-chip electroporation of HUVECs with pEGFP-N1 plasmids. (A) Bright-field and fluorescent micrographs of HUVECs after on-chip electroporation. Transfected cells expressed EGFP proteins (Green) and were stained with propidium iodide (Red). (B) Flow cytometry result of the transfected cells. The overall electroporation efficiency is 40.6% and the percentage of dead cells is 9.8%.

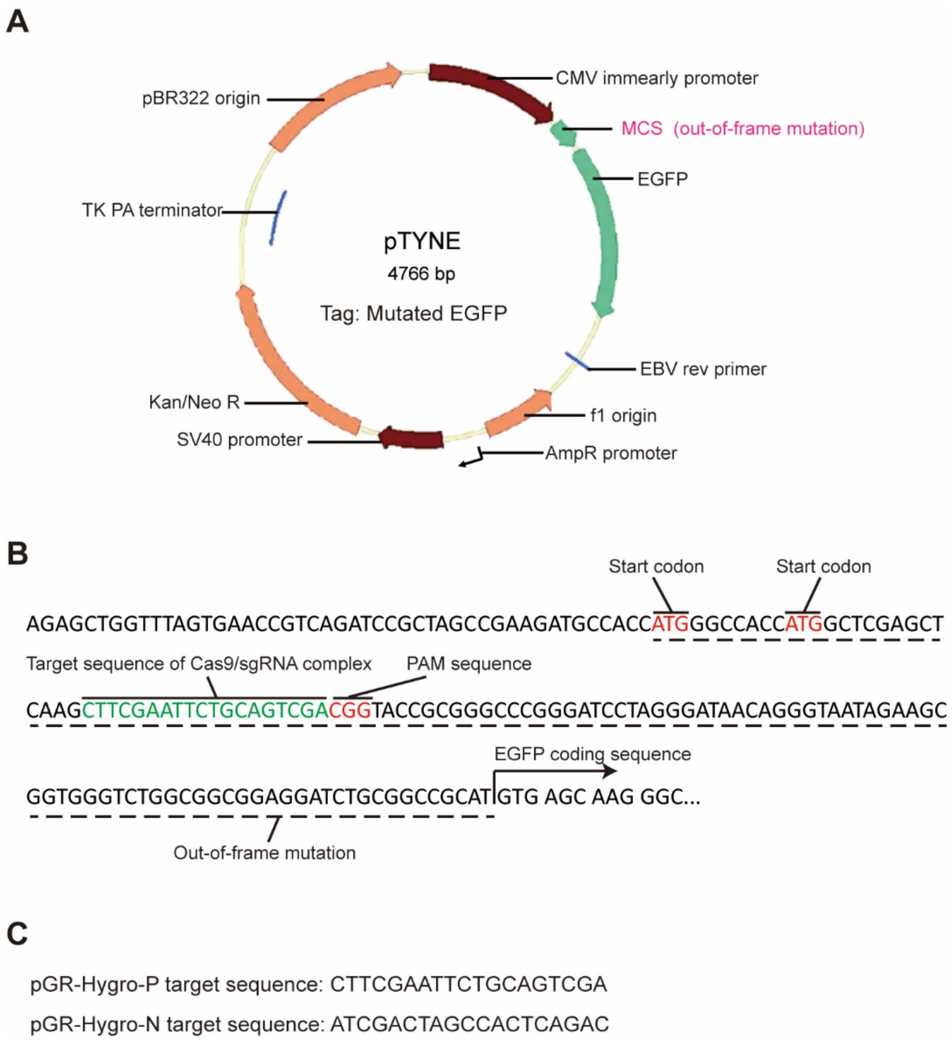

Figure S5. Schematic of the CRIPRE/Cas9 gene editing. (A) The structure of the pTYNE vector that contains an out-of-frame mutation between the start codons and the EGFP coding sequence. (B) The sequence between the CMV promotor and the EGFP. Two start codons are located 112-bp and 122-bp upstream from the EGFP sequence. Due to the out-of-frame mutation, the EGFP gene cannot express efficiently, leading to a very low level of green fluorescence. A sgRNA targeting the sequence (green) between the EGFP and the start codons can lead to short insertions or deletions by the non-homologous end joining (NHEJ), thus shift the reading frame of EGFP so that the protein can express correctly. (C) The target sequences of the pGR-Hygro-P and the pGR-Hygro-N sgRNA plasmids.

Supplementary video

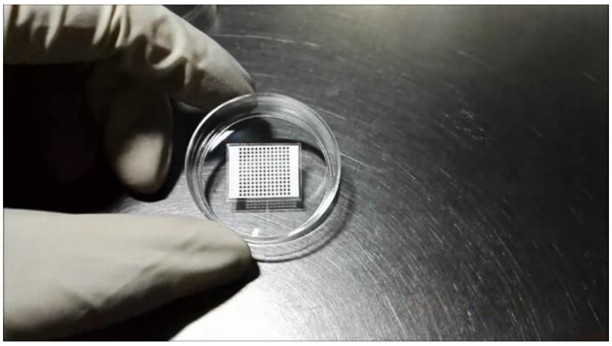

Video S1. Operation of the superhydrophobic microwell chip. The microchip was first submerged into a solution. After the excess solution outside the wells was aspirated out, a nano-liter droplet array in the microwells spontaneously formed due to the repelling effect of the superhydrophobic polymers to aqueous solutions.

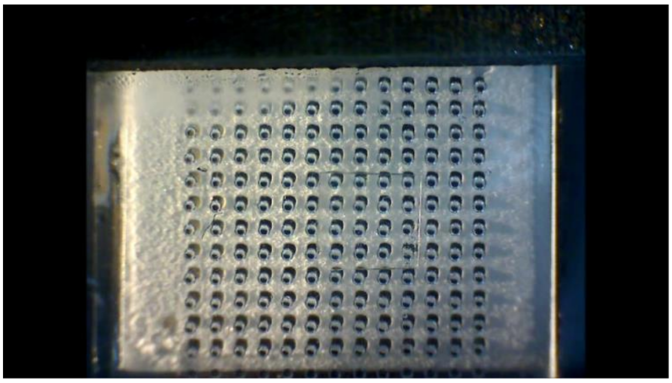

Video S2. Alignment of a spotted reagent array to a superhydrophobic microwell array viewed from the top of the chip assembly. With the aid of a CCD camera and a custom-built micromanipulator, these two arrays can be easily aligned together without any cross-contamination.

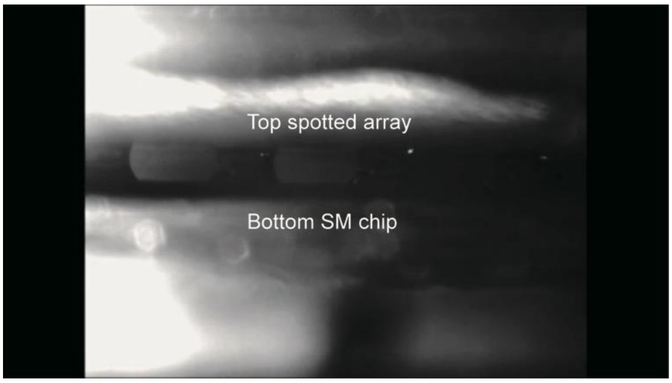

Video S3. Alignment of a spotted reagent array to a microwell array from a side view. Reagents were delivered into microwells through a droplet-to-droplet contact without any cross-contamination due to the superhydrophobic polymers.
